# Merkel cell polyomavirus-specific immune responses in patients with Merkel cell carcinoma receiving anti-PD-1 therapy

**DOI:** 10.1186/s40425-018-0450-7

**Published:** 2018-11-27

**Authors:** Natalie J. Miller, Candice D. Church, Steven P. Fling, Rima Kulikauskas, Nirasha Ramchurren, Michi M. Shinohara, Harriet M. Kluger, Shailender Bhatia, Lisa Lundgren, Martin A. Cheever, Suzanne L. Topalian, Paul Nghiem

**Affiliations:** 1 Department of Medicine, Divisions of Dermatology and Medical Oncology, University of Washington, 850 Republican Street, Seattle, WA 98109 USA; 2 Cancer Immunotherapy Trials Network, Fred Hutchinson Cancer Research Center, Seattle, WA USA; 3 Comprehensive Cancer Center, Section of Medical Oncology, Yale University School of Medicine, New Haven, CT USA; 4 Division of Medical Oncology, Department of Medicine, University of Washington, Seattle, WA USA; 5 Department of Surgery, Johns Hopkins University School of Medicine, and Johns Hopkins Bloomberg~Kimmel Institute for Cancer Immunotherapy, Baltimore, MD USA

**Keywords:** Merkel cell carcinoma, Merkel cell polyomavirus, Viral cancer antigen, Immunotherapy, Pembrolizumab, Anti-PD-1, T cell

## Abstract

**Background:**

Merkel cell carcinoma (MCC) is an aggressive skin cancer that frequently responds to anti-PD-1 therapy. MCC is associated with sun exposure and, in 80% of cases, Merkel cell polyomavirus (MCPyV). MCPyV-specific T and B cell responses provide a unique opportunity to study cancer-specific immunity throughout PD-1 blockade therapy.

**Methods:**

Immune responses were assessed in patients (*n* = 26) with advanced MCC receiving pembrolizumab. Peripheral blood mononuclear cells (PBMC) were collected at baseline and throughout treatment. MCPyV-oncoprotein antibodies were quantified and T cells were assessed for MCPyV-specificity via tetramer staining and/or cytokine secretion. Pre-treatment tumor biopsies were analyzed for T cell receptor clonality.

**Results:**

MCPyV oncoprotein antibodies were detectable in 15 of 17 (88%) of virus-positive MCC (VP-MCC) patients. Antibodies decreased in 10 of 11 (91%) patients with responding tumors. Virus-specific T cells decreased over time in patients who had a complete response, and increased in patients who had persistent disease. Tumors that were MCPyV(+) had a strikingly more clonal (less diverse) intratumoral TCR repertoire than virus-negative tumors (*p* = 0.0001).

**Conclusions:**

Cancer-specific T and B cell responses generally track with disease burden during PD-1 blockade, in proportion to presence of antigen. Intratumoral TCR clonality was significantly greater in VP-MCC than VN-MCC tumors, suggesting expansion of a limited number of dominant clones in response to fewer immunogenic MCPyV antigens. In contrast, VN-MCC tumors had lower clonality, suggesting a diverse T cell response to numerous neoantigens. These findings reveal differences in tumor-specific immunity for VP-MCC and VN-MCC, both of which often respond to anti-PD-1 therapy.

**Electronic supplementary material:**

The online version of this article (10.1186/s40425-018-0450-7) contains supplementary material, which is available to authorized users.

## Background

Merkel cell carcinoma (MCC) is an aggressive neuroendocrine skin cancer. The majority of MCCs are driven by the Merkel cell polyomavirus (MCPyV) T-antigen oncoproteins, while the remaining MCCs are ‘virus-negative’, and are driven by UV-induced mutagenesis [1–3]. Both virus-positive (VP) and virus-negative (VN) MCC are immunogenic and can elicit MCC-specific CD8+ and CD4+ T cell responses [4, 5], and most patients with VP-MCC mount a B cell response against the MCPyV T-antigen oncoproteins [6, 7]. MCC demonstrates immune escape by upregulating PD-1 in tumor infiltrating and peripheral blood MCPyV-specific T cells [8], which negatively modulates immune function in response to intratumoral expression of its major ligand, PD-L1 [9, 10]. Excitingly, a number of recent clinical trials have shown that approximately half of MCC patients, regardless of tumor viral status, experience durable responses after treatment with agents that block PD-(L)1 signaling [11–13]. However, to date there are no clear clinical or tumor characteristics that can predict which patients are more likely to respond [11, 12] and moreover, the mechanisms of response and resistance are poorly understood.

Virus-driven cancers such as MCC offer ideal model systems in which to track and assess cancer-specific T and B cell responses throughout treatment with PD-1 axis blockade as the majority of MCCs are driven by key portions of MCPyV oncoproteins. This facilitates study of shared antigens and thus tumor-specific T cell responses across VP-MCC patients via well-defined HLA-tetramers [4, 14], whereby T cells can be isolated without the use of activation markers allowing for the study of T cells that may become dysfunctional/unresponsive after chronic antigenic exposure. In addition, B cells express PD-1 [15] and MCC offers the unique opportunity to assess cancer-specific B cell activity throughout the therapeutic course by quantifying MCPyV-oncoprotein antibody titers. Lastly, insight may be gained by comparing T cell responses between responding patients with VP-MCC versus VN-MCC, as the latter are driven by less defined and likely more heterogeneous UV-induced neoantigens.

Characterization of cancer-specific immune responses in MCC may elucidate correlates of anti-PD-1 response/non-responsiveness, and direct us to agents which might be rationally combined with PD-1 inhibition for improved efficacy. These findings may be generalizable to other malignancies for which the ability to perform such detailed analysis is limited by lack of the tools described above. To address these goals, we analyzed tumor and peripheral blood samples from 26 patients receiving pembrolizumab through an ongoing clinical trial [11] to characterize the T and B cell responses to MCC over the course of therapy. Of the pre-treatment parameters we assessed, none offer a clinically reliable indicator to guide whether or not a patient’s tumors would respond to anti-PD-1. In contrast, we have identified MCPyV-specific T- and B- cell parameters that correlate with whether or not a patient’s tumors have responded to pembrolizumab. Additionally, we find a striking difference in the pre-treatment TCR clonality between virus-positive and virus-negative MCC tumors.

## Methods

### Patients and patient samples

All patients enrolled on this study provided written informed consent. Patients received pembrolizumab intravenously every 3 weeks at a dose of 2 mg/kg, for a maximum period of 2 years with radiologic assessment every 9 weeks [11]. Investigators reported clinical responses based on CT scans per RECIST 1.1, as follows: complete response (CR), partial response (PR), stable disease (SD) or progressive disease (PD*)* based on imaging collected from time of enrollment to 08/01/2016. An initial response must have been confirmed by a serial CT scan showing the same result to be considered a confirmed response [16]. Blood samples were drawn for correlative laboratory analyses at pre-treatment, 12 weeks after starting therapy, and at 9-week intervals thereafter. Peripheral blood mononuclear cells (PBMC) were cryopreserved after routine Ficoll preparation by a specimen processing facility at the Cancer Immunotherapy Trials Network.

### Determination of tumor MCPyV status

Tumor viral status was defined by expression of Large T-antigen within the tumor or by production of antibodies to small T-antigen as both are restricted to patients with MCPyV-positive tumors, as previously described [6, 17].

### Serology

Baseline serum samples from patients (*n* = 26) were used to determine if patients produced antibodies to the MCPyV small T-antigen oncoprotein as described [6] (Laboratory Medicine, University of Washington, Seattle, WA). Patients with titers above 74 standard titer units (STU) were considered positive and had subsequent time points measured for changes in oncoprotein antibody titer over the course of anti-PD-1 therapy.

### MCPyV-specific tetramer staining

All patients were HLA class I genotyped to determine eligibility for CD8 T cell specific MCPyV-tetramer screening (Bloodworks Northwest, Seattle, WA). PBMC collected from patients with HLA class I (HLA-I) types that corresponded to available MCPyV-specific tetramers (A*02:01, A*24:02, B*07:02, B*35:02, or B*37:01; *n* = 17 patients) were analyzed without knowledge of patient viral status. PBMC was analyzed using a previously optimized and standardized HLA-I tetramer staining protocol as follows: PBMC (> 2 × 10^6) at baseline and 12 weeks after starting therapy were stained with anti-CD8-FITC antibody (Clone 3B5, Life Technologies), 7-AAD viability dye (BioLegend), and appropriate APC or PE-labeled tetramers (Immune Monitoring Lab, Fred Hutchinson Cancer Research Center) and data collected on a FACSAriaII (BD Biosciences). FlowJo version 10.0.8 (TreeStar) was used for analysis and determination of the percentage of live cells in the tetramer, CD8, double positive region. Samples with > 0.01% of CD8+ T cells co-staining with tetramers were considered positive. For patients with tetramer(+) T cells, all subsequent PBMC obtained on trial were also analyzed.

### Circulating T cell response to MCPyV

Pre-treatment PBMC (*n* = 26) and post-treatment PBMC obtained at end of treatment (*n* = 3), 12 weeks (*n* = 14) or 21 weeks (n = 2) after initiating therapy were analyzed in intracellular cytokine secretion assays (HIV Vaccine Trials Network, Seattle WA). PBMC (10^6) were thawed and allowed to rest at 37C overnight before interrogation with four peptide pools containing 13aa-long, overlapping peptides (~ 25 peptides each) corresponding to the persistently expressed region of MCPyV T-antigens [4], as well as positive (CMV peptides) and negative (DMSO) controls, in presence of costimulatory antibodies and Brefeldin A as previously described [18, 19]. Cells were stained for a panel of markers including: CD3, CD4, CD8, IFN-γ and IL-*2* in addition to PD-1 (clone J105). Data were collected by flow cytometry on a LSRII and analyzed with FlowJo version 8.8.7 (TreeStar). Responsiveness to MCPyV peptides was based on IFN-γ and IL-2 expression by CD8+ and CD4+ T cells. Subjects with IFN-γ and/or IL-2 production upon MCPyV peptide pool stimulation were not further broken down due to restrictions on specimen availability.

### Tumor T cell receptor sequencing

Pre-treatment formalin-fixed paraffin-embedded (FFPE) tumor biopsy material (20–25 μm thick molecular curls or material scraped from pre-cut slides, *n* = 26) were submitted to Adaptive Biotechnologies for genomic DNA extraction of tissue, TCRβ sequencing and normalization as previously described [20]. Of the 26 tumor samples, 2 did not have enough TCR sequence reads for further analysis. To determine T cell receptor clonality, Shannon entropy was calculated on the estimated number of genomes (≥2) of all productive TCRs and normalized by dividing by the log2 of unique productive sequences in each sample. Clonality was calculated as 1- normalized entropy.

## Results

We assessed whether the presence of B or T cell reactivities against MCPyV T-antigens in patients with VP-MCC correlated with clinical outcomes. Data for serum positivity of oncoprotein-specific (T-antigen) antibodies, presence of MCPyV-specific tetramer+ CD8+ T cells, positivity of CD8+ T cell IFN-γ expression in response to MCPyV peptides and clinical response according to RECIST 1.1 is summarized for patients with VP-MCC tumors in Table 1. B cell and T cell reactivities to MCPyV T-antigens were not found in patients with VN-MCC tumors (data not shown).

**Table 1:** Pre-treatment virus-specific B and T cell reactivities in 17 patients with MCPyV-positive MCC receiving pembrolizumab

Patient no.	Antibodies to small T-antigen^a^	MCPyV tetramer analysis^b^	MCPyV intracellular cytokine reactivity^c^	Response assessed by RECIST 1.1^d^
3	+	+	–	CR
7	+	+	–	CR
8	+	+	+	PR
6	+	+	–	PR
9	+	+	–	PD
16	+	+	–	PR
12	+	–	–	PR
21	+	–	–	CR
19	+	–	+	PD
4	+	N/A	–	PR
13	+	N/A	–	PR
26	+	N/A	–	PR
23	+	N/A	–	PD
15	+	N/A	–	PD
25	+	N/A	–	PR
14	–	N/A	–	CR
10	–	–	+	PR

^a^ Baseline serum samples from all patients were used to measure MCPyV small T-antigen oncoprotein antibody titers at Laboratory Medicine (University of Washington, Seattle, WA) as described [6]. Titers above 74 STU were considered positive as negative control sera titers fall below 74 STU [7]

^b^ All patients were low-resolution HLA class I genotyped to determine eligibility for CD8 T cell specific MCPyV peptide-HLA class I tetramer screening (Bloodworks Northwest, Seattle, WA). Pre- and post-treatment peripheral blood mononuclear cells (PBMCs) collected from patients with HLA class I types that corresponded to available MCPyV-specific tetramers (A*02:01, A*24:02, B*07:02, B*35:02, or B*37:01; *n* = 17 patients) were stained with appropriate tetramers and analyzed by flow cytometry. Samples with > 0.01% of CD8+ T cells co-staining with tetramers were considered positive. N/A (Not Available): nine patients, regardless of tumor viral status, had HLA class I types not amenable to tetramer staining and could thus not be evaluated for the presence of T cells recognizing MCPyV

^c^ PBMCs pre-treatment and post-treatment blood collections (week 12 or 21) were stimulated with pools of MCPyV-specific peptides in a flow cytometry-based intracellular cytokine secretion assay (HIV Vaccine Trials Network, Seattle, WA). PBMCs that secreted interferon-gamma and/or IL-2 robustly (≥0.1% of CD8 T cells after background subtraction) were considered reactive to MCPyV

^d^ Abbreviations for RECIST 1.1 response criteria are as follows: *CR* complete response, *PR* partial response, *PD* progressive disease

### MCPyV-specific B cell responses track with tumor response to pembrolizumab

**Fig. 1:**
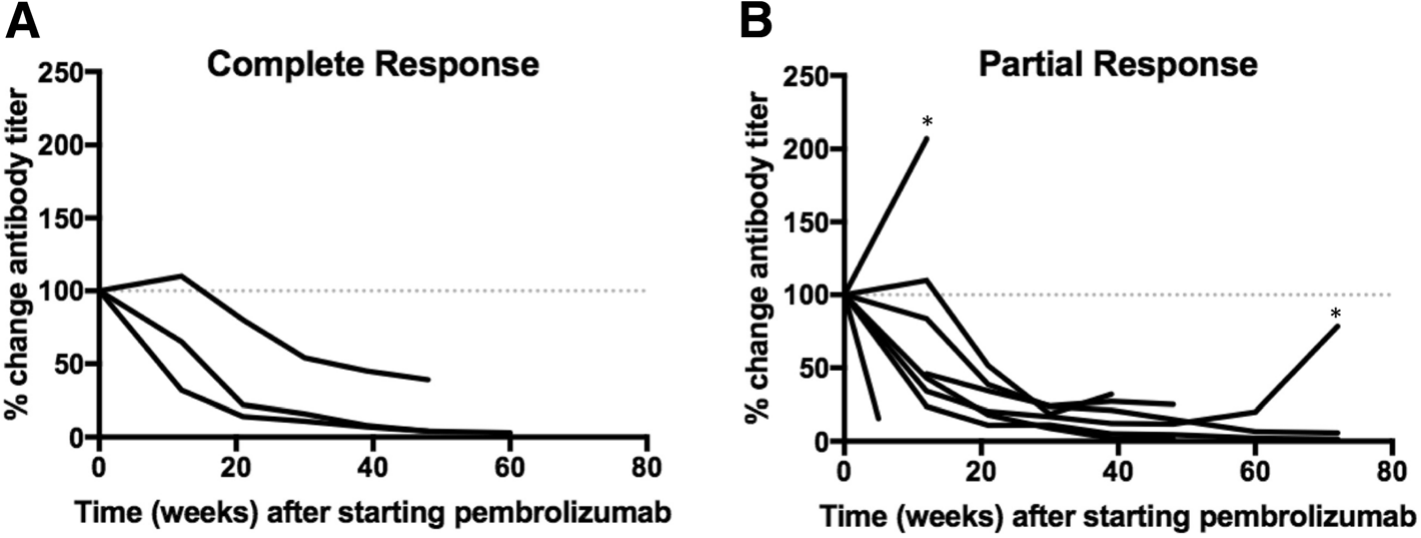
MCPyV-oncoprotein antibody titers over the course of anti-PD-1 therapy. 15 of 17 (88%) patients with VP-MCC tumors produced antibodies specific for MCPyV small T oncoprotein while no VN-MCC patients produced antibodies. MCPyV-oncoprotein antibody titer was tracked over time in seropositive individuals with available post-treatment serum samples (*n* = 13). Titers are plotted as percent change from baseline (100%). **a)** Patients with a complete response experienced a decrease in titer (*n* = 3). **b)** Among partial responders (*n* = 8), titer initially decreased over time in 7 of 8 patients. Two patients subsequently recurred (denoted by *); clinical detection of recurrence was preceded by a rise in titer in both cases

We measured B cell reactivity to MCPyV by quantifying serum titers against the small T-antigen oncoprotein, regardless of tumor viral status. Oncoprotein-specific antibodies have previously been found to be highly specific for patients with VP-MCC versus patients with VN- MCC or healthy controls. Furthermore, antibody titer has been shown to rise and fall with disease burden and to be a valuable tool to identify early recurrences [6, 7]. Oncoprotein antibodies were detected in pre-treatment serum from 15 of 17 patients with VP-MCC (88%) and 0 of 9 patients with VN-MCC (Table 1 and Additional file 1). Post-treatment serum samples were available from 20 of 26 patients. None of the seronegative patients developed oncoprotein antibodies after treatment initiation. Thirteen patients had detectable oncoprotein antibody titers that could be tracked over time. Overall, titers decreased significantly in those who completely or partially responded to therapy (Fig. 1). In addition, disease recurrence was associated with an increase in titer. Specifically, in two patients with an initial partial response, an increase in antibodies preceded clinically evident disease progression (Fig. 1b). For two patients who did not respond to pembrolizumab, antibody titers increased or remained stable (data not shown). Thus, patients treated with anti-PD-1, like those treated with other agents [6, 7], had oncoprotein antibody titers that tracked with disease burden.

### MCPyV-specific tetramer+ T cells

Tumor-specific CD8+ T cells may be activated and expanded by pembrolizumab to mediate anti-tumor effector functions. We therefore evaluated the presence and frequency of MCPyV-specific T cells throughout the therapeutic course using previously established HLA class I, MCPyV-specific tetramers (HLA restriction elements and epitopes summarized in Additional file 2 [4, 14]. Pre- and post-treatment PBMC from all patients with an HLA corresponding to at least one of five MCPyV-specific tetramers (*n* = 18 patients), were screened for MCPyV-specific CD8+ T cells, regardless of tumor MCPyV status. Tetramer(+), CD8+ T cells were detected among pre-treatment PBMC in 6 of 10 VP-MCCs (66%) versus 0 of 8 (0%) VN-MCCs.

**Fig. 2:**
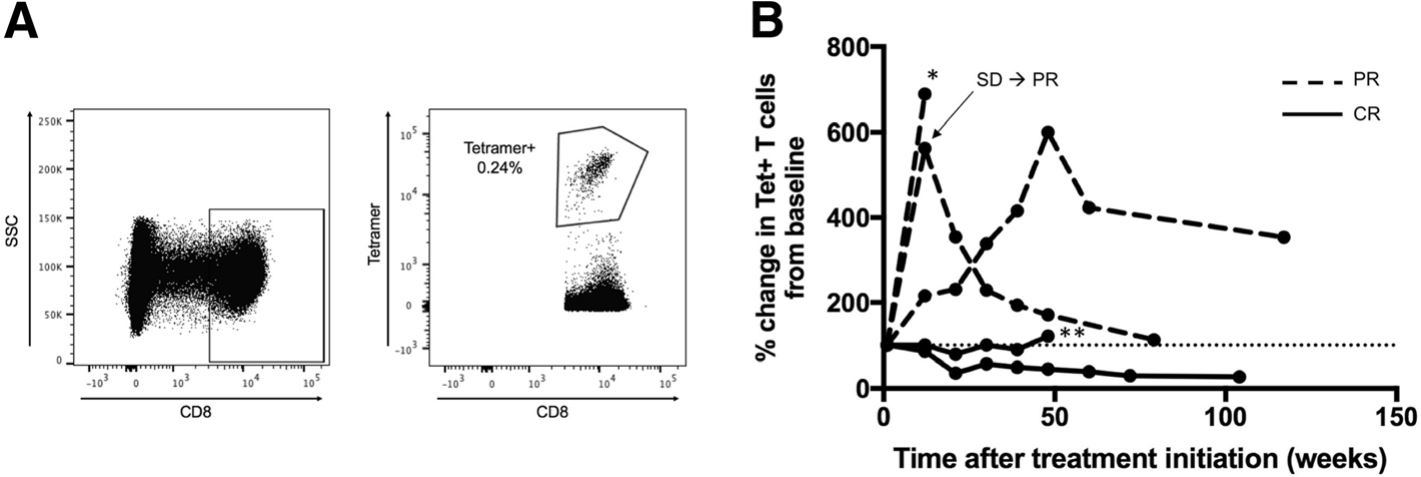
Frequency of MCPyV-specific CD8 T cells over the course of anti-PD-1 therapy. MCPyV-specific HLA class I tetramer-positive T cells were detected in pre-treatment PBMC in 6 of 9 (66%) of patients with VP-MCC tumors and appropriate HLA-I types, and in 0 of 8 patients with VN-MCC tumors with appropriate HLA class I types. **a)** Representative gating strategy for detection of MCPyV-specific T cells as indicated by tetramer binding. **b)** The frequency of tetramer-positive T cells increased after therapy in patients with a partial response (dashed, *n* = 3), yet remained similar to baseline or decreased in patients with a complete response (black, *n* = 2). Two patients subsequently recurred (denoted by * for recurrence on treatment and ** for recurrence after end of treatment)

MCPyV-specific T cells have previously been shown to increase in number with greater disease burden and decrease after treatment with surgery or radiation [8]. We hypothesized however, that pembrolizumab could instead induce proliferation of antigen-specific cells, increasing the number of MCPyV-specific T cells despite decreases in tumor burden. To test this, we determined the frequency of tetramer+ T cells in PBMC throughout the therapeutic course in patients with detectable T cells at baseline and with available post-treatment PBMC (*n* = 5 patients). For evaluable patients who had a complete response (*n* = 2), the frequency of tetramer+ T cells decreased or remained stable throughout the therapeutic course. In contrast, for patients with a partial response (*n* = 3) the frequency of MCPyV-specific T cells initially increased during therapy, but later fell as tumor burden decreased (Fig. 2 and Additional file 3).

### MCPyV-specific T cell activity

We also investigated whether circulating T cells became more reactive against MCPyV peptides after treatment with pembrolizumab. Pre- and post-treatment PBMC (19 patients) and pre-treatment only PBMC (7 patients) were stimulated with four pools of MCPyV peptides (13aa peptides of ~ 25 peptides per pool) corresponding to the persistently expressed region of MCPyV (see Methods and Additional file 2) [4]. Phenotypic lineage markers to denote CD8 T cells and cytokines (IL-2 and IFN-γ) were assessed via flow cytometry (Additional file 4). PBMC were tested without knowledge of patient’s tumor viral status.

**Fig. 3:**
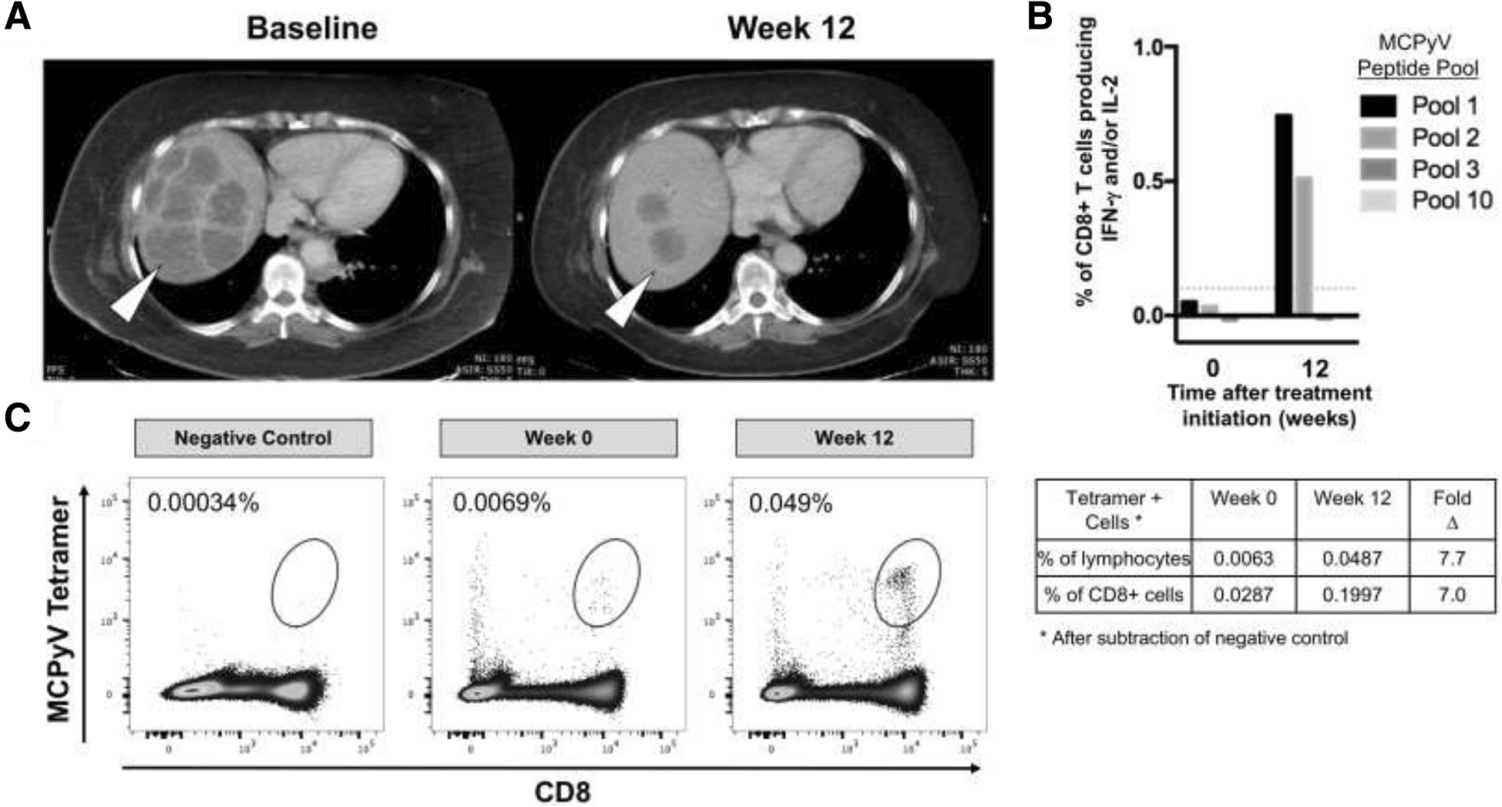
T cell reactivity to MCPyV-specific peptides increased after therapy in a patient who had a robust partial response to pembrolizumab. **a)** There was a significant reduction in burden of liver metastases (white arrow heads) as visualized by CT scans obtained at baseline and 12 weeks after initiating therapy. **b)** IFN-γ and IL-2 production by CD8+ cells from circulating PBMC to pools of MCPyV-specific peptides from samples obtained immediately pre-treatment and after 12 weeks of pembrolizumab therapy show a ~15x increase in anti-MCPyV-reactivity to peptide pools 1 and 2 after subtraction of background stimulation by DMSO. **c)** The frequency of tetramer+ CD8 cells restricted to HLA-B*07:02 ‘APNCYGNIPL’ (an epitope in Pool 1) increased significantly (~7x) after therapy

Anti-PD-1 staining did not show any association with cytokine response (data not shown). Additionally, no responses by CD4+ T cells were detected from any PBMC sample obtained pre- or post-treatment, as defined by production of IFN-γ and/or IL-2 at > 0.1% over DMSO-stimulated negative controls (data not shown). In contrast, CD8+ T cell responses to MCPyV peptides were detected in 3 of 17 patients with virus-positive tumors (responses defined as above). One patient had responses to one peptide pool of similar magnitude in both pre- and post-treatment PBMC. A second patient had a response in pre-treatment PBMC but post-treatment PBMC were unavailable. The third patient had partial tumor regression on pembrolizumab therapy, based on computed tomography (CT) scans obtained at baseline and 12 weeks after initiating therapy that revealed a significant reduction of necrotic liver masses (Fig. 3a). In parallel, this patient’s CD8+ T cell responses against two MCPyV peptide pools increased ~15x in the post-treatment PBMC (Fig. 3b). Tetramer(+) T cells recognizing the HLA-B*07:02 restricted epitope ‘APNCYGNIPL’ (present in a responsive peptide pool) increased ~ 7 fold in frequency post-treatment (Fig. 3c). It is possible that this dramatic increase in MCPyV-specific T cell number and effector function contributed to this patient’s tumor regression, though alternative explanations cannot be ruled out.

### TCR repertoire in pretreatment tumors

T cell receptor (TCR) clonality describes TCR diversity, wherein increased clonality reflects a reduced diversity of T cell clones within a population (i.e., intra-tumoral), presumably responding to a restricted number of antigens. In contrast, low clonality refers to a highly diverse T cell population, suggesting antigenic diversity or a large number of distinct clones specific for the same antigen. Increased clonality of the immune infiltrate within tumors is thought to represent an enrichment of cancer antigen-specific T cells and has been associated with improved response to pembrolizumab in patients with metastatic melanoma [21]. We sequenced the complementarity determining region 3 (CDR3) region of T cell receptor beta chain (*TRB*) of peri-tumoral and intratumoral T cells from pre-treatment tumor biopsy material (*n* = 24) and calculated the *TRB* clonality of each tumor.

**Fig. 4:**
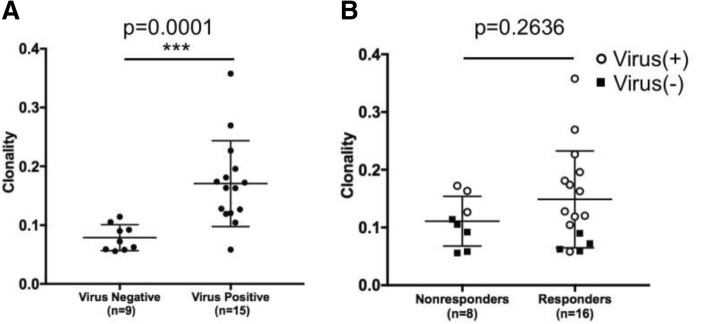
Comparison of T cell receptor clonality by viral status and response to anti-PD-1. **a**) TCR clonality is significantly higher in patients with VP-MCCs compared to those with VN-MCCs (*p* = 0.0001 by Mann-Whitney test). **b)** TCR clonality is not associated with response to pembrolizumab (*p* = 0.2636 by Mann-Whitney test). This observation remains true when comparing clonality among responding versus non-responding patients whose tumors are virus-positive (virus(+) = open circles; virus(−) = black squares)

There was no significant difference in tumoral TCR clonality between patients who did or did not respond to pembrolizumab (Fig. 4**,** *p* = 0.2636). However, TCR clonality was significantly increased in patients with virus-positive MCCs (*n* = 15) compared to those with virus-negative MCCs (*n* = 9) (Fig. 4**,** *p* = 0.0001).

## Discussion

Immunotherapy via blockade of the PD-1/PD-L1 pathway has recently become the standard of care for most patients with advanced MCC [22]. In this study of patients with metastatic MCC receiving the PD-1 blocking agent pembrolizumab [11], we have taken advantage of the unique viral etiology of most MCCs to explore MCPyV-specific humoral and cellular immune responses as an indicator of antitumor immunity.

Monitoring MCPyV oncoprotein antibodies in MCC has become a useful tool for clinical management because increases in antibody titer precede clinically evident recurrent disease [7] and this test is now included in the current MCC management guidelines in the US [22]. The role of B lymphocytes in cancer is complex as B cells can either enhance or inhibit anti-tumor immune responses (reviewed in Yuen) [23]. In addition, all B cell subsets express PD-1 [24, 25]. There is evidence that PD-1 axis blockade can increase antigen-specific antibody secretion [26] or reduce regulatory B cell function [27], supporting the hypothesis that these therapies could augment anti-tumor B cell responses. To our knowledge, our study is the first to assess cancer-specific B cell responses during PD-1 blockade. We found that among patients with clinical responses to pembrolizumab, oncoprotein antibody titers dropped as tumor burden decreased, suggesting that pembrolizumab did not augment tumor antigen-specific antibody production. In contrast, oncoprotein antibodies increased in patients whose disease did not respond to pembrolizumab (Fig. 1). These results suggest that the main determinant for oncoprotein antibody production is tumor volume (presumably linked to burden of tumor viral oncoproteins). Importantly, in two patients within this cohort who initially responded but later developed progressive disease, oncoprotein antibody titers increased prior to clinically evident disease progression, providing an early indication of acquired resistance to pembrolizumab. In one of these cases in which the target lesions responded, progressive disease developed within the central nervous system, a location that is not a common metastatic site for MCC and is not routinely imaged in MCC management [22]. This suggests tracking antibody titer may prompt earlier or more extensive imaging in patients with increasing titers.

Because relevant tumor antigens are largely unknown for many cancer types, study of tumor-specific T cells is typically not feasible. Previous reports suggest that effector cells are reinvigorated following successful anti-PD-1 therapy [28], but these studies are often performed on bulk T cell populations, regardless of antigen specificity. We quantitated MCPyV-specific T cells in the peripheral blood throughout the therapeutic course to understand how anti-PD-1 therapy affects the kinetics of tumor-specific CD8+ T cell expansion or contraction. The frequency of MCPyV-specific T cells remained stable or decreased over time for the two evaluable patients who experienced complete responses. By contrast, for all three evaluable patients with partial responses, MCPyV-specific T cells initially increased. Two patients had a sustained partial response and subsequent contraction of their MCPyV-specific T cell population over time (Fig. 2). Though these data are on a limited number of patients, they are in accord with previous trends of T cell responses tracking with tumor burden after effective surgery or radiation [8]. One possible explanation for the difference in frequency of MCPyV-specific CD8+ T cells between patients who experienced a CR versus those who had a PR may have been due to rapid clearance of tumor antigens in patients with complete radiologic regression and subsequent contraction of MCPyV-specific T cell populations before the first on-therapy blood draw at 12 weeks. In contrast, continued antigenic burden in patients with PR may have continuously stimulated T cell expansion. An analogous study of cancer-antigen specific T cells in non-small cell lung cancer showed neoantigen-specific CD8+ T cell frequency increased 3 weeks after initiation of PD-1 blockade, in parallel with tumor regression, and fell shortly thereafter [29]. Unfortunately, the earliest blood collection time point on the study described here was 12 weeks post-treatment and we suspect that early transient increases in T cell number were likely missed. When assessing T cell function, rather than frequency, we rarely observed MCPyV-specific T cell cytokine responses in the peripheral blood of MCC patients regardless of response to pembrolizumab. Indeed, MCPyV-specific T cell activity increased in only one of 12 patients with VP-MCC clinically responding to pembrolizumab (Fig. 3). Potential explanations for the lack of response in the other 11 patients include: (i) the 12 week time point may have been too late to observe transiently augmented T cell reactivity; (ii) MCPyV-specific T cells did not gain function after anti-PD-1 and remained unable to secrete effector cytokines; (iii) T cells were stimulated with 13-aa MCPyV oncoprotein peptides and different length peptides may produce additional responses; and (iv) relevant immune responses may be directed at non-viral antigens including UV-neoantigens.

Enrichment of T cell clones within a tumor suggests infiltration and expansion of tumor-antigen specific T cells. This can be assessed by TCR sequencing. Indeed, increased TCR clonality within pretreatment tumors has been associated with response to pembrolizumab in melanoma [21]. However, this was not observed in our study of an equivalent number of MCC patients. This may be due to variability within tumor specimens, or may indicate that TCR clonality and response to PD-1 are not as clearly associated in MCC. Importantly, VP-MCC tumors had a markedly higher clonality when compared to VN-MCC tumors (Fig. 4**,** *p* = 0.0001). We hypothesize that VP-MCCs elicit a strong immune response through the expression of a small number of highly immunogenic viral antigens, resulting in recruitment of a limited number of unique T cells that expand once in the tumor. Conversely, VN-MCCs often have large numbers of UV-induced mutations that lead to expression of mutated self-proteins expected to be immunogenic [2, 30]. The large number of mutations in VN-MCCs may result in recruitment of a diverse T cell population and apparently lower clonality. Indeed, there exists a positive correlation between increased TCR diversity and higher mutational load in both mouse and human cancers [31, 32].

There are several limitations to this study, most notably the small sample size. Though we were able to measure B and T cell responses in the peripheral blood, the earliest post-therapy blood draw in our study design was 12 weeks after initiation of anti-PD-1 therapy. Clinical responses to anti-PD-1 in MCC arose more rapidly than anticipated. It is likely that key immune responses such as transient increases in B or T cell number or function may have been missed because no blood was collected prior to week 12 [28, 33]. Additionally, there were 5 validated available HLA-I MCPyV peptide tetramers available for this study. Of the 17 patients with VP-MCC, 9 had HLA-I types allowing one or more tetramers to be used. We attempted to overcome this limitation by assessing production of IFN-γ, IL-2 and other cytokines following peptide stimulation to identify additional MCPyV-specific T cell responses. However, we infrequently observed peptide specific cytokine production in this assay, possibly due to the inability of dysfunctional T cells to secrete cytokines. Therefore, our analyses captured what was likely only a subset of existing MCPyV-specific T cell responses.

## Conclusions

There is a great need to determine mechanisms of response to anti-PD-1 therapy across all tumor types. We made use of the unique immunogenic VP-MCC and VN-MCC subtypes of this cancer to gain insight into B and T cell responses to tumor antigens that is not feasible for most cancers. However, we did not identify any immune correlates of clinical response that could be used as predictive biomarkers to determine which patients should receive pembrolizumab. Instead, our findings demonstrate that MCPyV-specific B and T cell responses typically track with tumor burden, regardless of therapeutic modality: pembrolizumab or traditional therapy with surgery and/or radiation [6, 8]. Our results support the prognostic value of monitoring MCPyV oncoprotein antibodies in patients with advanced MCC who are at greater risk for recurrent disease. The strikingly greater intratumoral T cell clonality found in VP-MCCs highlights differences in immune response to MCPyV versus UV-neoantigens in the two MCC subtypes. Further studies of these MCC-specific immune responses should advance our understanding of which patients are most likely to respond and also, which agents might be best rationally combined with anti-PD-(L)1 to improve patient outcomes.

## Additional files

Additional file 1: Serology results. Raw results for MCPyV-specific oncoprotein antibodies were reported as Standard Titer Units (STUs). Patient with a value of 75 or greater were considered oncoprotein antibody producers and values below 74 were considered negative. Abbreviations for RECIST 1.1 response criteria are as follows: CR = complete response; PR = partial response; PD = progressive disease. (DOCX 114 kb)

Additional file 2: Class I HLA Tetramers and MCPyV peptide pools. Description of data: **A)** Summary of HLA and peptide combinations used for CD8 class I tetramers including which position of the MCPyV oncoprotein (small, common, or large T antigen) the peptide corresponds to. **B)** Schematic of MCPyV peptide pools and locations of tetramer epitopes. Details of peptide pools are available in Iyer et al., 2011. (DOCX 364 kb)

Additional file 3: Frequency of tetramer+ CD8 T cells. Frequency of MCPyV tetramer positive CD8 T cells are reported in percent of all CD8s with background subtracted. Abbreviations for RECIST 1.1 response criteria are as follows: CR = complete response; PR = partial response; PD = progressive disease. (DOCX 69 kb)

Additional file 4: Frequency of IFN-γ and/or IL-2 secreting CD8 T cells in response to Merkel polyomavirus peptide pools. IFN-γ and/or IL-2 in **A)** 2 of 13 VP-MCC responders and **B)** 1 of 4 VP-MCC non-responders was detectible via flow cytometry after a 16 h stimulation with MCPyV peptide pools. Dotted line represents background signal cutoff. *Post = first blood draw after initiation of treatment. (DOCX 425 kb)

## Abbreviations

CR: Complete response
CT: computed tomography
FFPE: Formalin-fixed paraffin-embedded
HLA-I: Human leukocyte antigen class I
IFN-γ: Interferon gamma
IL-2: Interleukin 2
MCC: Merkel cell carcinoma
MCPyV: Merkel cell polyomavirus
PBMC: Peripheral blood mononuclear cells
PD: Progressive disease
PD-1: Programmed cell death protein 1
PD-L1: Programmed death-ligand 1
PR: Partial response
RECIST: Response Evaluation Criteria in Solid Tumors
SD: Stable disease
STU: Standard Titer Units
TCR: T cell receptor
UV: Ultraviolet
VN-MCC: Virus-negative Merkel cell carcinoma
VP-MCC: Virus-positive Merkel cell carcinoma
