# Supplementary material for: Merkel cell polyomavirus-specific immune responses in patients with Merkel cell carcinoma receiving anti-PD-1 therapy
Source: J Immunother Cancer. 2018 Nov 27;6:131. doi: 10.1186/s40425-018-0450-7 (PMC6258401; doi:10.1186/s40425-018-0450-7)
Supplement: Supplementary file 1 — Serology results. Raw results for MCPyV-specific oncoprotein antibodies were reported as Standard Titer Units (STUs). Patient with a value of 75 or greater were considered oncoprotein antibody producers and values below 74 were considered negative. Abbreviations for RECIST 1.1 response criteria are as follows: CR = complete response; PR = partial response; PD = progressive disease. (DOCX 114 kb) [file 40425_2018_450_MOESM1_ESM.docx]

**Additional file 1: Serology results**

|  | Patient no. | Week 0 | Week 12 | Week 21 | Week 30 | Week 39 | Week 48 | Week 60 | Week 72 | End of treatment |
| --- | --- | --- | --- | --- | --- | --- | --- | --- | --- | --- |
| Sero-positive | | | | | | | | | |  |
| CR | 3 | 63,100 | 41,100 | 13,600 | 10,300 | 4,840 | 2,650 | 2,190 |  |  |
|  | 21 | 32,100 | 35,200 | 25,800 | 17,400 | 14,400 | 12,500 |  |  |  |
|  | 7 | 25,100 | 8,100 | 3,600 | 2,640 | 1,840 | 953 |  |  |  |
|  | | | | | | | | | | |
| PR | 4 | 575,000 | 480,500 | 222,500 | 136,000 | 122,000 | 86,100 | 37,600 | 32,900 |  |
|  | 6 | 865,000 | 204,000 | 93,000 | 96,600 | 38,300 | 18,400 | 13,800 |  |  |
|  | 13 | 116,000 | 50,000 | 20,900 | 10,200 | 2,240 | 2,000 |  |  |  |
|  | 26 | 35,900 | 16,500 | 12,300 | 8,620 | 9,730 | 8,980 |  |  |  |
|  | 25 | 2,300 |  |  |  |  |  |  |  | *359 (Wk 5) |
|  | 8 | 90,900 | 186,000 |  |  |  |  |  |  |  |
|  | 12 | 118,000 | 40,200 | 24,000 | 19,800 | 14,500 | 13,800 | 23,400 | 92,600 |  |
|  | 16 | 260,000 | 285,500 | 134,000 | 47,800 | 83,000 |  |  |  |  |
|  | | | | | | | | | | |
| PD | 23 | 105,000 |  |  |  |  |  |  |  | **106,000 (Wk 5) |
|  | 9 | 69,700 |  |  |  |  |  |  |  | **92,900 (Wk 5) |
|  | 15 | 96,300 |  |  |  |  |  |  |  |  |
|  | 19 | 1,380,000 |  |  |  |  |  |  |  |  |
| Sero-negative | | | | | | | | | | |
|  |  |  |  |  |  |  |  |  |  |  |
| CR | 17 | <74 | <74 |  |  |  |  |  |  |  |
|  | 14 | <74 |  |  |  |  |  |  |  |  |
|  | | | | | | | | | | |
| PR | 2 | <74 | <74 |  |  |  |  |  |  |  |
|  | 5 | <74 | <74 |  |  |  |  |  |  |  |
|  | 10 | <74 | <74 |  |  |  |  |  |  |  |
|  | 20 | <74 | <74 |  |  |  |  |  |  |  |
|  | 22 | <74 | <74 |  |  |  |  |  |  |  |
|  | | | | | | | | | | |
| SD | 11 | <74 |  |  |  |  |  |  |  |  |
|  | | | | | | | | | | |
| PD | 1 | <74 | <74 |  |  |  |  |  |  |  |
|  | 24 | <74 |  |  |  |  |  |  |  |  |
|  | 18 | <74 |  |  |  |  |  |  |  |  |

Raw results for MCPyV-specific oncoprotein antibodies were reported as Standard Titer Units (STUs). Patient with a value of 75 or greater were considered oncoprotein antibody producers and values below 74 were considered negative. Abbreviations for RECIST 1.1 response criteria are as follows: CR = complete response; PR = partial response; PD = progressive disease.

* Patient 25 went off study due to an adverse event and no further samples were collected; however, the patient subsequently experienced a CR.

** Patients 9 and 23 went off study due to progressive disease and no further samples were collected.
