# Supplementary material for: Merkel cell polyomavirus-specific immune responses in patients with Merkel cell carcinoma receiving anti-PD-1 therapy
Source: J Immunother Cancer. 2018 Nov 27;6:131. doi: 10.1186/s40425-018-0450-7 (PMC6258401; doi:10.1186/s40425-018-0450-7)
Supplement: Supplementary file 2 — Class I HLA Tetramers and MCPyV peptide pools. Description of data: A) Summary of HLA and peptide combinations used for CD8 class I tetramers including which position of the MCPyV oncoprotein (small, common, or large T antigen) the peptide corresponds to. B) Schematic of MCPyV peptide pools and locations of tetramer epitopes. Details of peptide pools are available in Iyer et al., 2011. (DOCX 364 kb) [file 40425_2018_450_MOESM2_ESM.docx]

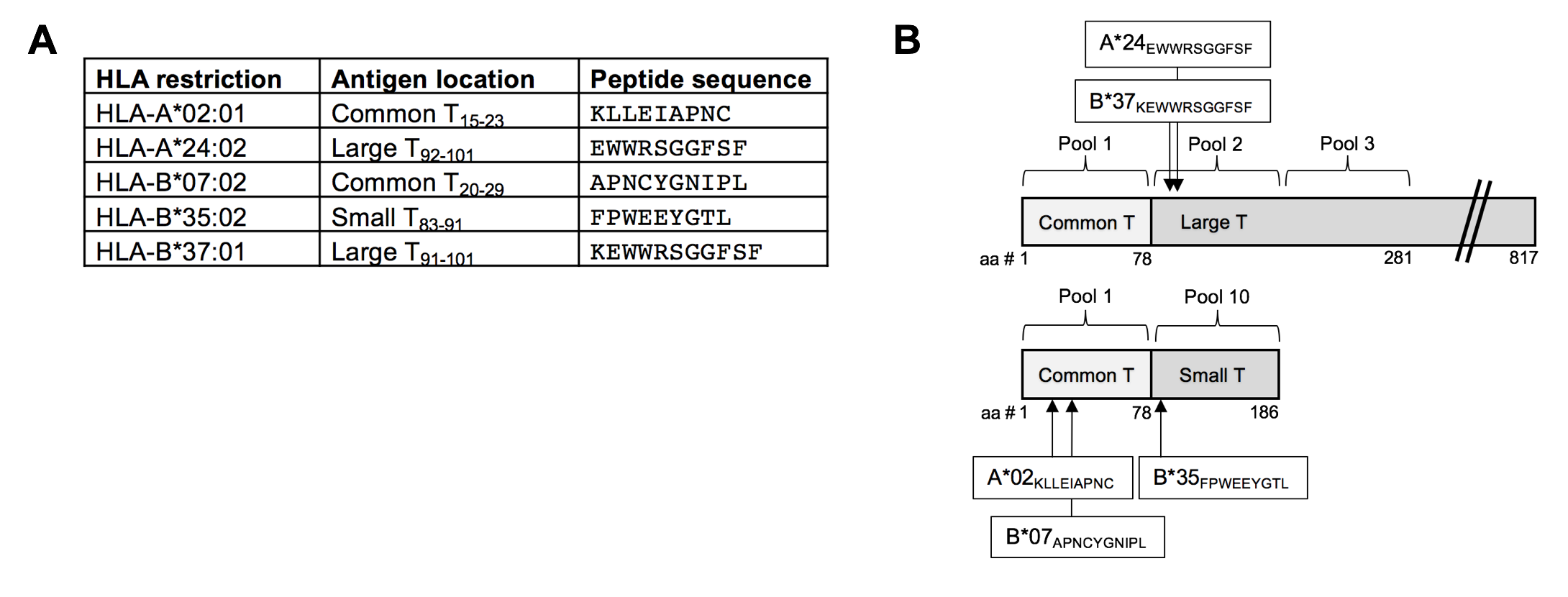


**Additional file 2: Class I HLA Tetramers and MCPyV peptide pools.**

**A)** Summary of HLA and peptide combinations used for CD8 class I tetramers including which position of the MCPyV oncoprotein (small, common, or large T antigen) the peptide corresponds to. **B)** Schematic of MCPyV peptide pools and locations of tetramer epitopes. Details of peptide pools are available in Iyer et al., 2011.

**References:**

1. Iyer JG, Afanasiev OK, McClurkan C, Paulson K, Nagase K, Jing L, et al. Merkel cell polyomavirus-specific CD8(+) and CD4(+) T-cell responses identified in Merkel cell carcinomas and blood. *Clinical Cancer Research.* 2011;17(21):6671-6680.
