# Supplementary material for: Merkel cell polyomavirus-specific immune responses in patients with Merkel cell carcinoma receiving anti-PD-1 therapy
Source: J Immunother Cancer. 2018 Nov 27;6:131. doi: 10.1186/s40425-018-0450-7 (PMC6258401; doi:10.1186/s40425-018-0450-7)
Supplement: Supplementary file 3 — Frequency of tetramer+ CD8 T cells. Frequency of MCPyV tetramer positive CD8 T cells are reported in percent of all CD8s with background subtracted. Abbreviations for RECIST 1.1 response criteria are as follows: CR = complete response; PR = partial response; PD = progressive disease. (DOCX 69 kb) [file 40425_2018_450_MOESM3_ESM.docx]

**Additional file 3: Frequency of tetramer+ CD8 T cells**

|  | Patient no. | Week 0 | Week 12 | Week 21 | Week 30 | Week 39 | Week 48 | Week 60 | Week 72 | Week 79 | Week 104 | Week 117 |
| --- | --- | --- | --- | --- | --- | --- | --- | --- | --- | --- | --- | --- |
|  | | | | | | | | | | | | |
| CR | 3 | 0.280 | 0.240 | 0.100 | 0.160 | 0.137 | 0.126 | 0.110 | 0.083 |  | 0.076 |  |
|  | 7 | 0.019 | 0.019 | 0.015 | 0.019 | 0.017 | 0.023 |  |  |  |  |  |
|  | | | | | | | | | | | | |
| PR | 6 | 0.013 | 0.028 | 0.030 | 0.044 | 0.054 | 0.078 | 0.055 |  |  |  | 0.046 |
|  | 8 | 0.029 | 0.200 |  |  |  |  |  |  |  |  |  |
|  | 16 | 0.048 | 0.270 | 0.170 | 0.110 | 0.093 | 0.082 |  |  | 0.054 |  |  |
|  | | | | | | | | | | | | |
| PD | 9 | 1.240 |  |  |  |  |  |  |  |  |  |  |

Frequency of MCPyV tetramer positive CD8 T cells are reported in percent of all CD8s with unstained background subtracted. Control specimens with known tetramer positive frequencies were used in parallel for quality control assessment for each tetramer type as well as non-HLA-matched MCC patients to control for non-specific background staining. Abbreviations for RECIST 1.1 response criteria are as follows: CR = complete response; PR = partial response; PD = progressive disease.
