# Supplementary material for: Merkel cell polyomavirus-specific immune responses in patients with Merkel cell carcinoma receiving anti-PD-1 therapy
Source: J Immunother Cancer. 2018 Nov 27;6:131. doi: 10.1186/s40425-018-0450-7 (PMC6258401; doi:10.1186/s40425-018-0450-7)
Supplement: Supplementary file 4 — Frequency of IFN-γ and/or IL-2 secreting CD8 T cells in response to Merkel polyomavirus peptide pools. IFN-γ and/or IL-2 in A) 2 of 13 VP-MCC responders and B) 1 of 4 VP-MCC non-responders was detectible via flow cytometry after a 16 h stimulation with MCPyV peptide pools. Dotted line represents background signal cutoff. *Post = first blood draw after initiation of treatment. (DOCX 425 kb) [file 40425_2018_450_MOESM4_ESM.docx]

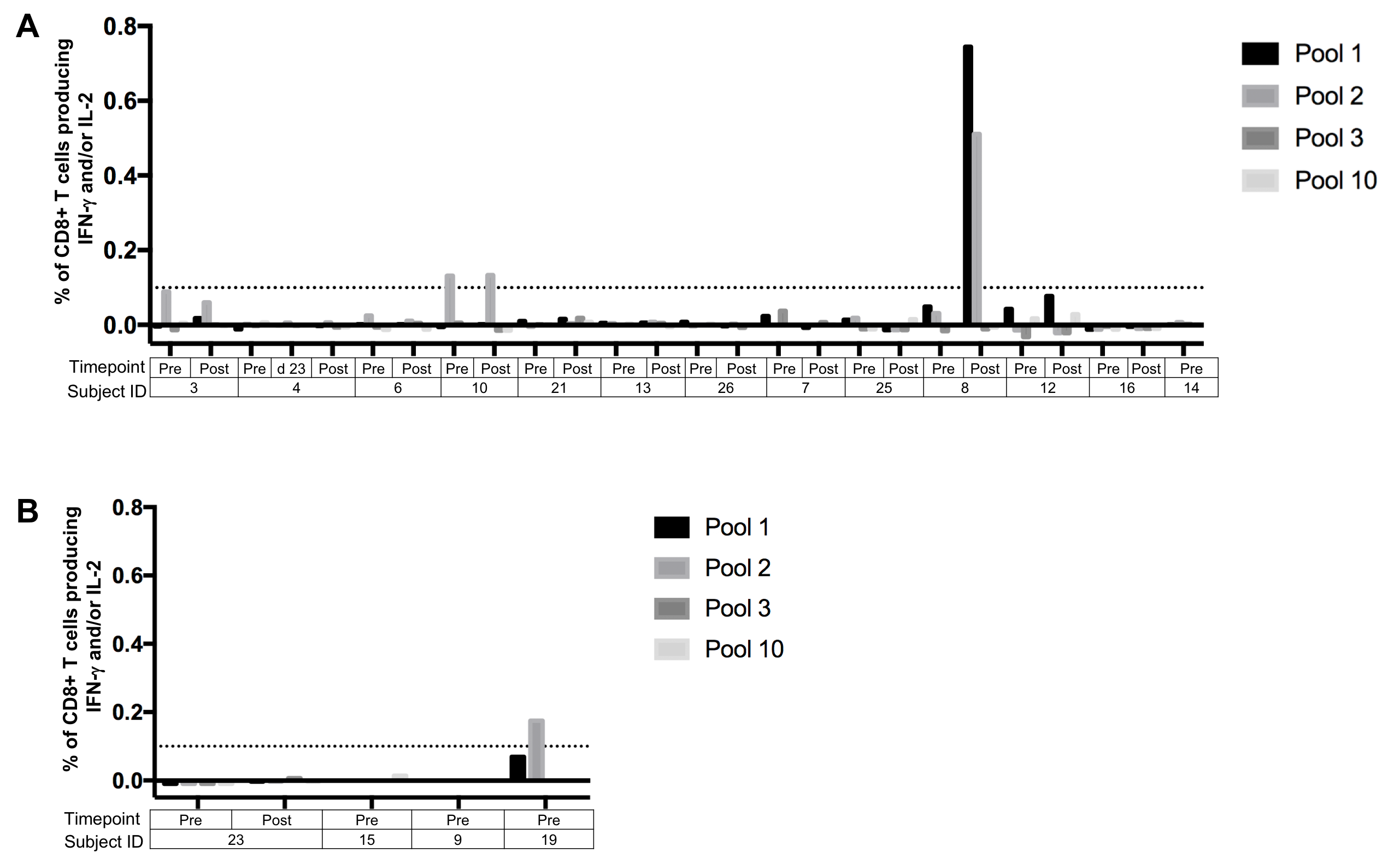


**Additional file 4: Frequency of IFN**γ **and/or IL-2 secreting CD8 T cells in response to Merkel polyomavirus peptide pools**.

IFNg and/or IL-2 in **(A)** 2 of 13 VP-MCC responders and **(B)** 1 of 4 VP-MCC non-responders was detectible via flow cytometry after a 16 hour stimulation with MCPyV peptide pools. *Dotted line represents background signal cutoff. *Post = first blood draw after initiation of treatment*
